# Facilitators and barriers of routine psychosocial distress assessment within a stepped and collaborative care model in a Swiss hospital setting

**DOI:** 10.1371/journal.pone.0285395

**Published:** 2023-06-30

**Authors:** Nicola Julia Aebi, Iris Baenteli, Günther Fink, Gunther Meinlschmidt, Rainer Schaefert, Matthias Schwenkglenks, Anja Studer, Sarah Trost, Sibil Tschudin, Kaspar Wyss, the SomPsyNet Consortium

**Affiliations:** 1 Swiss Center for International Health, Swiss Tropical and Public Health Institute, Allschwil, Switzerland; 2 University of Basel, Basel, Switzerland; 3 Department of Psychosomatic Medicine, University Hospital and University of Basel, Basel, Switzerland; 4 Epidemiology and Public Health, Swiss Tropical and Public Health Institute, Allschwil, Switzerland; 5 Division of Clinical Psychology and Cognitive Behavioural Therapy, International Psychoanalytic University Berlin, Berlin, Germany; 6 Division of Clinical Psychology and Epidemiology, Department of Psychology, University of Basel, Basel, Switzerland; 7 Institute of Pharmaceutical Medicine (ECPM), University of Basel, Basel, Switzerland; 8 Division of Prevention, Department of Health Canton Basel-Stadt, Basel, Switzerland; 9 Department of Geriatric Medicine FELIX PLATTER, Basel, Switzerland; 10 Department of Obstetrics and Gynecology, University Hospital and University of Basel, Basel, Switzerland; University of Sharjah, UNITED ARAB EMIRATES

## Abstract

**Background:**

Stepped and Collaborative Care Models (SCCMs) have shown potential for improving mental health care. Most SCCMs have been used in primary care settings. At the core of such models are initial psychosocial distress assessments commonly in form of patient screening. We aimed to assess the feasibility of such assessments in a general hospital setting in Switzerland.

**Methods:**

We conducted and analyzed eighteen semi-structured interviews with nurses and physicians involved in a recent introduction of a SCCM model in a hospital setting, as part of the SomPsyNet project in Basel-Stadt. Following an implementation research approach, we used the Tailored Implementation for Chronic Diseases (TICD) framework for analysis. The TICD distinguishes seven domains: guideline factors, individual healthcare professional factors, patient factors, professional interactions, incentives and resources, capacity for organizational change, and social, political, and legal factors. Domains were split into themes and subthemes, which were used for line-by-line coding.

**Results:**

Nurses and physicians reported factors belonging to all seven TICD domains. An appropriate integration of the psychosocial distress assessment into preexisting hospital processes and information technology systems was the most important facilitator. Subjectivity of the assessment, lack of awareness about the assessment, and time constraints, particularly among physicians, were factors undermining and limiting the implementation of the psychosocial distress assessment.

**Conclusions:**

Awareness raising through regular training of new employees, feedback on performance and patient benefits, and working with champions and opinion leaders can likely support a successful implementation of routine psychosocial distress assessments. Additionally, aligning psychosocial distress assessments with workflows is essential to assure the sustainability of the procedure in a working context with commonly limited time.

## Introduction

The global burden of mental disorders remains high [1]. Mental disorders often remain undetected or untreated, particularly in patients with mental–somatic multimorbidities [2, 3]. One possibility to overcome this gap are stepped and collaborative care models (SCCMs). The main idea of stepped care is to identify and deliver the least invasive, but most effective treatment, and then stepping up treatment if disease burden reaches a specific threshold [4]. Collaborative multi–professional care has been shown to be an important element for appropriately handling mental [5, 6] and somatic illnesses [7] in patients with mental–somatic multimorbidities.

SCCMs combine these two concepts and have been introduced in various countries and health care settings. The heterogeneity of the SCCMs implemented in a range of countries coupled to contextual specificities, does currently not allow concluding on standard and best implementation modalities of SCCMs for managing mental health problems. Yet, a successful embedment in routine health service provision requires a range of aspects to be considered as observed in implementation research. Implementation research gives insights into the factors affecting the implementation in a real world setting [8]. For instance, in a German project a major difficulty to implement a SCCM model within primary care was collaboration across a regionally wide-spread network [9]. Co-location of somatic and mental health specialists in the same working place [10, 11] is valuable to reach integrated care [12] and can help handling mental health conditions in somatic settings. Further, general hospitals in the United Kingdom successfully implemented routine depression and anxiety screenings in some specialties [13]. However, structural factors, such as the ward organization or staff availability, were factors impeding the mental health screening [13].

Here, we report insights from a recent project conducted in a general hospital setting in Switzerland. SomPsyNet is a healthcare project for SOMatic inpatients to prevent PSYchosocial stress consequences by establishing a stepped and collaborative care NETwork in the canton of Basel-Stadt [14, 15]. The SCCM implemented by SomPsyNet aims to improve the quality of life of patients with mental–somatic multimorbidities [14, 15]. First, ward physicians and nurses as well as patients themselves independently assess the patient’s psychosocial distress using a distress thermometer, which has been adapted from the commonly used distress thermometer in oncology [16]. The score is recorded in each patient’s electronic file; hospital information technology (IT) systems were adapted accordingly. Second, all patients are comprehensively screened for depressive, anxiety, and distressing somatic symptoms using validated and reliable assessment tools. The two-step screening shall be completed within the first 72 hours of hospitalization. If patients are distressed according to the two-step screening, they are offered a psychosomatic consultation providing them clinical assessment and appropriate treatment recommendations according to stepped care. To support the implementation of the first step of the SCCM, each ward had one face-to-face training. Additionally, an online training course was created to introduce SomPsyNet and the SCCM to healthcare professionals.

Organizational changes and the introduction of new systems such as the SCCM are challenging regarding various aspects. Two systematic reviews have summarized structural, financial, and individual barriers to integrating mental health in primary care settings [10, 17]. Leadership, reimbursement, and motivation represented important determinants of integration success [10, 17]. Yet, evidence of the facilitators and barriers encountered when SCCMs are implemented in hospital settings is lacking. Thus, we aimed to assess the facilitators and barriers of the psychosocial distress assessment implementation, which represents the first step of the SCCM in Basel-Stadt. We structured this study around a framework widely used in implementation research, the Tailored Implementation for Chronic Diseases (TICD) framework.

## Methods

### Study setting

Three hospitals in the canton of Basel-Stadt started the SomPsyNet study in 2020. While the University Hospital Basel and the University Department of Geriatric Medicine FELIX PLATTER are public hospitals including close collaboration with teaching and research, the Bethesda Hospital is a private hospital focusing on gynecology, rheumatology, and rehabilitation. With 37,108 inpatients being discharged in 2020 [18], the University Hospital Basel is the largest participating hospital. In comparison, the University Department of Geriatric Medicine FELIX PLATTER and the Bethesda Hospital had 5,143 and 6,108 discharged inpatients, respectively, in 2020 [19, 20].

### Study sample

The study sample consisted of nurses and physicians of all participating hospitals working in different specialties: internal medicine, gynecology, rehabilitation, rheumatology, and geriatric rehabilitation / acute geriatrics. To get insights into the facilitators and barriers of the SomPsyNet implementation, nurses and physicians differing in age and gender participated in the interviews. Sampling relied on a purposive sampling representing health professionals with different socio-demographic characteristics and holding different roles and responsibilities in respect to the SCCM. All interviewees participated in the SomPsyNet implementation. After a first set of interviews in 2020 conducted to evaluate the perceived importance of and experiences with mental health in hospital settings focusing on somatic health conditions [21], NJA contacted the same interviewees in 2021 by email (N = 18). Interviewees who did not work on a ward implementing the SCCM anymore or were not interested to be re-interviewed were replaced with new interviewees suggested by hospital ward line managers. Given the high time pressure in the hospital settings studied, we decided to conduct individual interviews rather than focus groups as assembling up to ten doctors or nurses for focus group discussions was not feasible.

### TICD framework

Following an implementation research approach, the TICD framework by Flottorp et al. [22] was used to clearly structure reported facilitators and barriers of the psychosocial distress assessment within the SCCM. This framework contains 57 potential determinants, which are grouped into seven domains: guideline factors, individual healthcare professional factors, patient factors, professional interactions, incentives and resources, capacity for organizational change, and social, political, and legal factors. These determinants are based on a literature review of twelve checklists, representing a comprehensive checklist to facilitate implementation research. The TICD framework was initially established and validated for health service interventions focusing on patients with chronic diseases in primary healthcare, but has also been applied in acute care settings [23] and long-term care [24] covering various diseases including mental health [25, 26].

### Data collection

The interview guide was based on the TICD framework (see S1 Text). Before starting the interviews, NJA (female epidemiologist with experience in qualitative research; PhD Candidate working for the external evaluation team) pilot tested the interview guide with a former member of the SomPsyNet project team. Between 26 May 2021, and 2 September 2021, NJA conducted all semi-structured interviews, in either Swiss German or German, depending on the interviewees’ preferences. The interviews took place face-to-face at the workplace of the interviewee or via video-communication due to the COVID-19 pandemic, depending on the interviewee’s preference. To the best of our knowledge, no other people were around during the interviews. NJA conducted interviews until no new information emerged. The interviews were audio-recorded and transcribed verbatim.

### Data analysis

**Fig 1:**
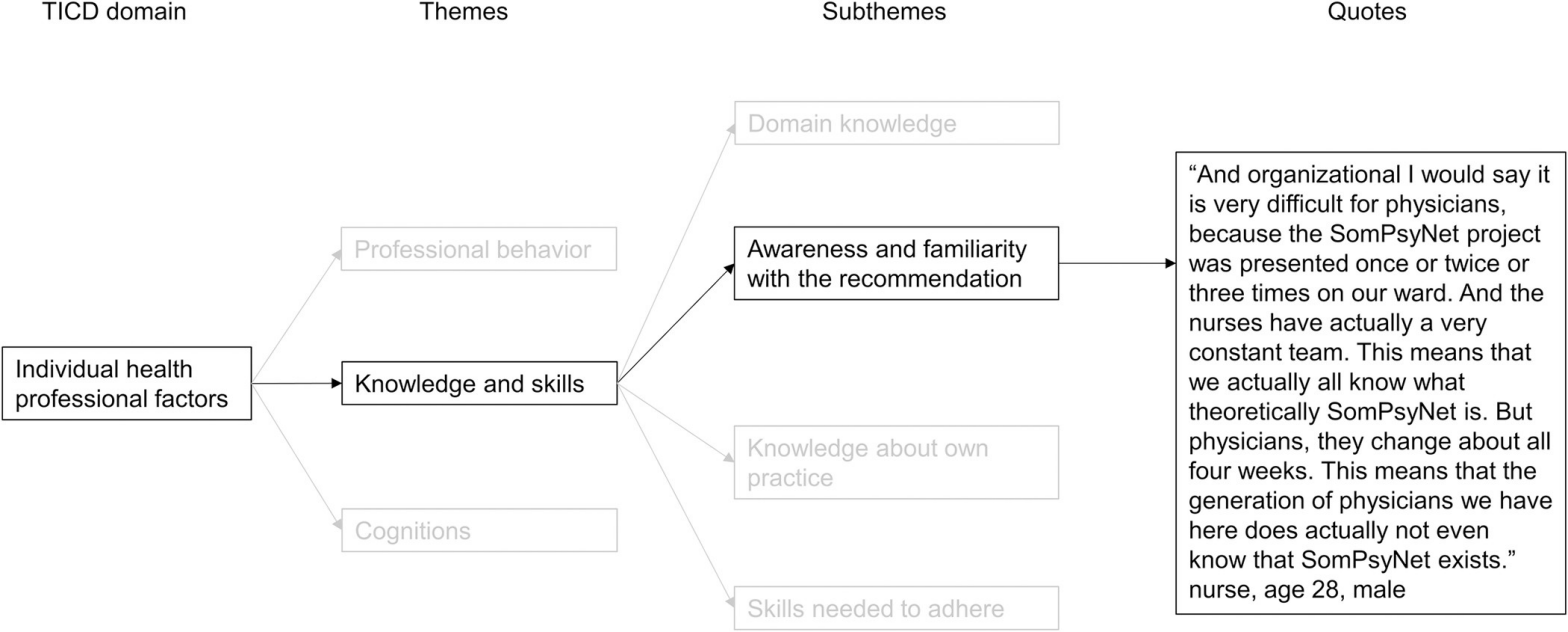
Example of the analysis applying the TICD framework.

Content analysis was done in NVivo 12 [27] using the framework method by Gale et al. which conceives the analysis of qualitative analysis along seven steps: 1) transcription, 2) familiarization with the interview, 3) coding, 4) developing a framework, 5) applying the framework, 6) charting data into a framework matrix, and 7) interpreting the data [28]. After transcription of the interviews, NJA familiarized herself with the content by multiple reading of the transcripts. Then, NJA deductively coded the interviews by applying the TICD framework. The TICD domains were split into determinants (themes and subthemes) that were used for line-by-line coding. Decisions were made using the definitions of TICD determinants provided by Flottorp et al. [22]. Fig 1 displays an example of the subtheme “compatibility” within the TICD domain “professional factors”. Example quotes for each TICD determinant mentioned by the interviewees are presented in S1 Table. Afterwards, the data was charted in a data matrix. A summary of each TICD determinant (column) was written for each interviewee (row) and linked to illustrative quotes. This data matrix helped to interpret the data and write a memo for each TICD domain including the identified facilitators and barriers. Additionally, detailed researcher notes and discussion with NJA’s supervisor (KW) supported the analysis and interpretation of the interviews and thus, the rigor of the research, during the analysis. Due to the limited time resources of the interviewees, we did not share the transcripts or findings with the interviewees. NJA translated the example quotes from German to English. The Consolidated Criteria for Reporting Qualitative Research (COREQ-32) guided the reporting [29].

### Ethical considerations

The Ethics Committee Northwest and Central Switzerland (Ethikkomission Nordwest- und Zentralschweiz; EKNZ) approved this implementation study (ID Req-2019-01219). All interviewees gave written informed consent.

## Results

Ten nurses and eight physicians participated in the interviews. Three nurses knew the interviewer (NJA) from a first interview in 2020 while 15 interviewees, replacing others who could not be re-interviewed, did not know her. Table 1 shows average interviewee characteristics. The mean age of the interviewed nurses was higher than the one of the physicians, which translates into a longer professional experience. Further, we conducted more interviews with female than with male nurses who worked in various departments at the three included hospitals. The interviews lasted on average 29 min (range 20–44 min).

**Table 1:** Demographic characteristics of the interviewees, affiliated institutions, and interview duration (N = 18).

Characteristics of interviewees	Physicians (*n* = 8)	Nurses (*n* = 10)
Age [years]		
Mean (standard deviation = SD)	29.6 (3.5)	37.3 (10.8)
Range	26–35	23–56
Sex		
Female	4	8
Male	4	2
Time in profession [years]		
Mean (SD)	1.8 (1.1)	12.1 (8.9)
Range	0.75–4	4–34
Affiliated institution		
University Hospital Basel	3	3
University Department of Geriatric Medicine FELIX PLATTER	2	3
Bethesda Hospital	3	4
Department		
Rehabilitation/Rheumatology	1	2
Internal Medicine	3	3
Gynecology	2	1
Geriatrics	2	4
Interview duration [minutes]		
Mean (SD)	25.1 (4.3)	33.2 (8.0)
Range	20.4–32.2	22.1–44.1

Interviewees mentioned facilitators and barriers in all seven TICD domains. Capacity for organizational change and social, political, and legal factors were discussed less comprehensively than factors from other domains. Table 2 presents an overview of the facilitators and barriers cited by the interviewees. We provide a summary of all TICD domains in the following paragraphs. Additionally, S1 Table shows example citations of each determinant.

**Table 2:** Overview of facilitators and barriers within the Tailored Implementation for Chronic Diseases (TICD) domains.

TICD domain	Facilitators	Barriers
Guideline factors	• alignment to existing process • accessibility in IT system • well-tailored to most patients	• subjectivity of assessment • missing observability of benefits • time-consuming patient discussions
Individual healthcare professional factors	• sufficient knowledge and skills about psychosocial distress • no change to routine processes • adaptability to daily routines possible	• missing awareness of healthcare professionals about the SCCM high turnover / fluctuation of physicians • subjectivity impedes personal motivation
Patient factors	• value holistic care approaches	• missing capability to express specific patient needs • changes of emotional well-being during hospital stay
Professional interactions	• underlined team work • no change in referral processes between wards and psychosomatic services	• interest of senior physicians • alignment to other healthcare professionals’ assessment
Incentives and resources	• required resources are available • financial benefits for patients and healthcare system (efficiency gains)	• absence and insufficiency of continuing training offers • missing reminders about execution of assessment • missing objective tool
Capacity for organizational change	• supportive leadership • feedback on adherence	• missing feedback on correctness of assessment • low priority of assessment within routine procedures
Social, political, and legal factors	• interest of various stakeholders	• stigma of mental health conditions

### Guideline factors

The guideline factors domain covers information on the guideline’s clarity, feasibility, compatibility, and effort. The psychosocial distress assessment was perceived to be well integrated into the daily routines, including the preexisting IT system. Interviewees described the assessment as a good complement to existing tools focusing more on somatic health in general hospitals. Interviewees considered the psychosocial distress assessment within the SCCM worthwhile except for those patients staying only shortly in the hospital, where patient safety is of highest priority (e.g., surgery patients).

*“But I think with women who have recently given birth*, *I get to personal things much more quickly […]*. *With other patients who have surgery*, *I do not have to explain and say and talk that much*. *There*, *I rather make sure that the safety is guaranteed*.*”* nurse, age 49, female

The psychosocial distress assessment also faced several barriers falling in this domain. In contrast to the entire SCCM, the psychosocial distress assessment was mentioned to lack an evidence-base. It was perceived to rely on personal rating, or “gut feeling”. Together with the missing ability to observe immediate benefits for patients, healthcare professionals’ motivation to assess psychosocial distress was limited. Still, they indicated their sensitivity and awareness to mental health conditions among patients had increased. Additionally, discussions with patients about their physical, emotional, and social well-being was thought to be time consuming. Indeed, the SCCM implied additional tasks for physicians and nurses in a context of already preexisting high workload. Some interviewees mentioned that they had appraised patients’ mental health without using a structured approach and a particular score prior to the SCCM introduction. Therefore, the additional workload is limited:

*“Well*, *basically*, *we as gynecologists–of course*, *this is special–we are always involved*, *psychosocially as well*. *So*, *this means that not much changed for us*.*”* physician, age 29, male

### Individual healthcare professional factors

The individual healthcare professional factors domain provides information on healthcare professionals’ knowledge, skills, attitudes, and behaviors related to the guideline use. The general knowledge on reasons and consequences of psychosocial distress and skills of healthcare professionals (e.g., empathy, conversational skills, attention to recognize psychosocial distress signs and symptoms) facilitated the psychosocial distress assessment. Its integration into the admission interview with patients allowed assessing possible correlates for psychosocial distress without major changes in routines. On some wards, minor changes supported the integration of the psychosocial distress assessment into daily routine (e.g., electronic notes in patient file).

*“[…] and we make notes that this is already done*. *This means*, *everybody sees that it is already done*. *And if the note is missing*, *then*, *you know that you still have to do it and particularly pay attention to it in patient care*.*”* nurse, age 46, female

However, the perceived subjectivity in judgments within the assessment tool impeded the healthcare professionals’ motivation to assess patients’ psychosocial distress, leading to improper scoring of psychosocial distress. This was emphasized by the reported uncertainty about the accuracy of the healthcare professionals’ assessment. Further, interviewees lacked information about the SCCM. Interviewees mentioned that the high turnover rate of physician negatively affected the awareness of the assessment. Various physicians only learned about the SCCM including the psychosocial distress assessment accidentally without a more comprehensive understanding of its rationale.

*“Initially*, *all of us were a bit annoyed that we also have to do this in addition and do not know for what and why*. *This is an issue that you do not ‘SomPsyNet–ah cool’*, *but ‘again one more’*. *Simply because we do not have background information*.*”* physician, age 26, female

### Patient factors

Patient factors, such as their needs, behavior, or motivation, play an essential role in guideline implementation. According to most interviewees, the psychosocial distress assessment depended on the patient’s personality. Interviewees described that patients value holistic care and are open to principles and ideas of the SCCM, which increased healthcare professionals’ motivation to assess psychosocial distress.

Nevertheless, the assessment of psychosocial distress was difficult to conduct with introvert patients, patients with cognitive impairments, or language barriers. Interviewees indicated that changes in the patients’ well-being might depend on the day or time healthcare professionals assess the patient’s psychosocial distress.

*“[…] you see a patient for a day and the patient maybe he is having a good or a bad day*. *And then*, *you*, *as physician*, *give any number*. *And I feel that this can vary quite a bit*.*”* physician, age 29, female

### Professional interactions

Overall, the professional interactions domain is particularly interested in the team and referral processes. Already before the start of the SCCM, interprofessional collaboration was part of daily routines, especially in two smaller hospitals where good communication was highlighted. For instance, interprofessional meetings or collaborations with social workers or mental health specialist were reported to be helpful. Referral processes (e.g., between wards and psychosomatic medicine) were already in place and did not change with the SCCM introduction.

Nonetheless, mainly resident physicians mentioned that they were influenced by their senior physician’s interest in psychosocial distress. If seniors are not interested and do not recognize the relevance of this area, juniors do not adhere to the psychosocial distress assessment.

*“Maybe it depends on the senior physician one is working with*. *If he/she is open for such things or not*.*”* physician, age 35, male

Although nurses and physicians do not exchange views on the assessment as such, they stated that they sometimes adjust their assessment to the one who assessed the patient’s psychosocial distress first.

### Incentives and resources

Any incentives and resources like education, equipment, financial and human resources affecting the guideline implementation are part of the incentives and resources domain. Whereas resources such as IT systems and necessary support were available, this was not the case regarding the time needed to interact with patients. Additionally, interviewees mentioned that patients and the healthcare system at large could benefit financially from the systematic patient assessment because this could enable better psychosocial distress identification and management, leading to better treatment outcomes. Healthcare professionals themselves do not have financial or reputational incentives for assessing psychosocial distress.

Still, interviewees reported major barriers regarding the incentives and resources domain. While nurses would like to receive repeated trainings to raise awareness on the model again, physicians’ main criticism was the lack of training in general. This might be related to the physicians’ turnover rate, which is causing physicians to miss out the online training offered on the SCCM. Reminders could raise awareness in the IT system, which was proposed by some interviewees. Last, interviewees emphasized the preference for an objective assessment. Receiving specific questionnaires or checklists including what to look for would help to increase the quality of the psychosocial distress assessment and the motivation to carry out the assessment.

### Capacity for organizational change

The capacity for organizational change domain covers the capacity to implement changes in a specific setting. Interviewees emphasized the importance of supporting leadership, be it by the hospital or ward managers. Some ward managers increase awareness and motivation through personal reminders. Receiving feedback on coverage and correctness of the psychosocial distress assessment by the SomPsyNet project team or the hospital management could enhance healthcare professionals’ awareness and motivation further.

*“[…] Or like an interim analysis like ‘hey*, *you did a great job*, *somehow of the 300 patients you have cared for in the last five weeks*, *20% were completed*. *The goal is to accomplish 40% until I come back in three weeks*. *And one can somehow see the progress a bit*.*”* nurse, age 28, male

Healthcare professionals mentioned no consequences in the instance of missing assessments. This mirrored the perceived low priority of the psychosocial distress assessment within somatic medicine, which reflects an important barrier.

*“Well*, *I believe that if one thinks with the biopsychosocial model*: *if one improves the psychosocial side*, *then*, *the biological*, *somatic side will improve automatically*. *And I think*, *unfortunately*, *we ignore this a bit in the somatic medicine*.*”* physician, age 31, male

### Social, political, and legal factors

The social, political, and legal factors domain includes determinants outside the respective setting needed to strive for changes like the implementation of a psychosocial distress assessment. Interviewees mentioned stigma related to poor mental health negatively affected the SCCM implementation. However, influential people like politicians, the funders, the project team, and external mental health specialists might be interested in the early detection and adequate psychosocial distress treatment to improve patients’ quality of life, reduce health care costs, and establish a network of various important stakeholders.

## Discussion

This implementation study explored different facilitators and barriers related to the introduction and operation of a psychosocial distress assessment by physicians and nurses within a SCCM in a Swiss general hospital setting. While integrating the assessment in preexisting IT systems and daily processes (e.g., admission interview) at the hospital supported the introduction, major barriers were identified in the domains of guideline factors, individual healthcare professional factors, and incentives and resources.

Integrating patients’ psychosocial distress assessment into preexisting hospital processes and IT systems reduced additional staff efforts. Integrated IT systems also fostered the collaborative care implementation in somatic health settings in previous studies [11]. The possibility to tailor processes to the specificities of wards may increase the motivation to assess patients’ psychosocial distress. Adaptability enhances commitment, and thus, sustainability [30]. Further, leadership support fosters commitment of staff members [11] as observed in our study.

Interviewees questioned the usefulness of a psychosocial distress assessment tool that is based on subjective judgment and “gut feeling”, although intuition plays an important role in clinical settings [31], especially for nurses [32]. The intuitive conclusions need to be corroborated by objective assessments as proposed by a previous study [31], which is important, as shown in delirium research, where subjective assessments are associated with misclassifications [33]. This explains the interviewees’ desire to increase objectivity and standardization with examples, checklists, or questionnaires and thus, support the psychosocial distress assessment.

Additionally, insufficient knowledge about and awareness of mental health has been described in several studies [17, 21]. Mainly physicians mentioned lack of awareness of and familiarity with the psychosocial distress assessment and the entire SCCM. One important reason for particularly junior physicians highlighting this may be their frequent rotation in general hospitals, which made adequate information about the assessment and the SCCM a challenge. This may hinder its sustainability as observed in another implementation study in mental health [34].

Lastly, time constraints are widespread when implementing integrated care [10, 17, 35], and healthcare professionals face many competing priorities. Typically, the focus on somatic health is to the detriment of mental health conditions [10, 17, 21]. The psychosocial distress assessment was perceived to be of low priority in general hospitals, negatively affecting the SCCM implementation including the patient assessment. This might be reinforced by missing observability of the assessment’s benefits on healthcare professionals’ daily work or the senior physician’s potential lack of interest.

## Strengths and limitations

This study adds value to implementation science in general hospital settings by highlighting important facilitators and barriers of a time-constraint setting that have to be accounted for when implementing routine psychosocial distress assessment. The inclusion of three different hospitals and different wards increases the generalizability of the findings. However, the findings may not apply to wards with short hospital stays, such as surgical wards or emergency departments.

Some limitations need to be considered when interpreting the findings. First, the recruitment strategy may have led to limited views on the SCCM and its first step, the psychosocial distress assessment. The view of participating nurses and physicians may differ from other healthcare professionals’ views. Nonetheless, the interviewees mentioned major critical factors. Second, interviewees may give socially desirable answers. Letting healthcare professionals choose the interview location and mentioning the opportunity to suggest improvements may have reduced this bias. Further, some interviews were of comparatively short duration due to multiple competing tasks of the interviewees, limiting the possibility to cover topics of interest in-depth. To counteract this, we shared topics of interest and the interview guide ahead of the interview. Third, not the entire SCCM had been implemented at the time of the interviews. Only one physician had initiated a consultation with a mental health specialist because of the SCCM, at the time of the interview. Therefore, we can only conclude on the subjective psychosocial distress assessment by the healthcare professionals and not on subsequent consultations for mental conditions. Fourth, this study only includes the healthcare professionals’ opinions and not those of patients, mental health specialists, or hospital management. Insights on the patient factors, capacity for organizational change, and social, political, and legal factors domains are limited. Finally, we used a deductive approach, which allowed structuring the analysis of facilitators and barriers as observed by other implementation research. This reduces the likelihood of identifying new themes. However, the analysis captured new factors such as the turnover rate relevant in acute care settings already mentioned by others [23].

## Implications for practice and future research

Based on the Expert Recommendations for Implementing Change (ERIC) compilation [36], we propose several approaches to overcome the three most important barriers observed in our psychosocial distress assessment implementation as part of an SCCM in general hospital settings.

Checklists and examples should be made available so to make psychosocial distress assessments more consistent across healthcare professionals. For the assessment to be sustainable, healthcare professionals need to be aware of the benefits to their patients and why a psychosocial distress assessment is necessary [10, 30, 37], even if it is subjective. This can be achieved through regular personal training, e.g., offered for healthcare professionals starting a new position, particularly important in settings with a high turnover rate. The training should clearly demonstrate the evidence base and the required action to assess psychosocial distress. Clear guidance coupled with repeated training may promote standardized approaches and thus, reduce subjectivity in the assessment. Additionally, readily available online training should be easily accessible, for instance by linking the training to the assessment in the patient file. Educational strategies are widely used [38] and seem to positively affect care, patient health, and health systems in nursing [39] and particularly when implementing collaborative care into somatic health care settings [11]. Further, reminders integrated directly to the IT system increase the awareness and save time.

In this time sensitive setting, the interviewees reported low priority of the patient’s psychosocial distress assessment. Especially physicians emphasized thereby the importance of the senior physician’s interest in mental health. Senior physicians should act as champions or opinion leaders who support the implementation and positively influence the SCCM uptake. Other research saw that physician champions helped the implementation of collaborative care into primary care [10].

The results of this study were shared and discussed with the SomPsyNet project team. They will complement quantitative data, e.g., the percentage of patients who were assessed for psychosocial distress by physicians and nurses, and will altogether support tailoring the SomPsyNet intervention to implementation realities. For instance, the provision of the psychosocial distress assessment could be restricted to only one healthcare professional.

Future research should focus on three main aspects. First, staff turnover was important in an emergency department study using the TICD [23]. We agree with the authors that this is a major determinant in a hospital setting in general. Future implementation research should therefore focus on how implementation can be guaranteed despite high turnover rates and how barriers related to the turnover rate can be sustainably overcome to better recognize mental health and see SCCM as part of hospital operations.

Second, we found that literature on how healthcare professionals perceive subjective assessments of patients’ mental health is scarce. While our findings show that objective assessments are preferred to subjective ones, we suggest investigating experiences of different healthcare professionals in different settings to better understand the reasons for this.

Third, other stakeholders’ perspectives are essential, particularly those of patients. Other research has found that patients wish to separate mental and somatic health spatially [11, 40]. An additional barrier for psychosocial distress assessment was the perceived stigmatization [41]. These factors may impede the mental health project implementation in general hospitals. Hence, research should focus on patients’ experiences with and preferences about integrating mental health into somatic settings.

## Conclusion

Subjectivity of the assessment, lack of awareness due to high turnover rates, and low priority of the assessment due to time constraints posed major challenges when implementing a psychosocial distress assessment of patients admitted to general hospitals, especially for physicians. We suggest providing regular training for new employees, providing feedback on healthcare professional performance and the observed patient benefits, and appointing champions or opinion leaders to address these challenges in implementing a routine assessment for mental health in general hospitals. Furthermore, the integration of the psychosocial distress assessment into current hospital processes and systems is a necessary factor to facilitate the implementation in a time-constrained setting.

## Supporting information

S1 Text Interview guide: Psychosocial distress assessment. (DOCX)

S1 Table Facilitators and barriers according to the TICD domains, themes, and subthemes, illustrated by representative quotes. (DOCX)

## List of abbreviations

COREQ: Consolidated Criteria for Reporting Qualitative Research
ERIC: Expert Recommendations for Implementing Change
IT: information technology
SCCM: stepped and collaborative care model
SomPsyNet: comprehensive healthcare project for patients from SOMatic hospitals that promotes the prevention of PSYchosocial distress by establishing a stepped and collaborative care NETwork in Basel, Switzerland
TICD: Tailored Implementation for Chronic Diseases
