# Supplementary material for: Facilitators and barriers of routine psychosocial distress assessment within a stepped and collaborative care model in a Swiss hospital setting
Source: PLoS One. 2023 Jun 30;18(6):e0285395. doi: 10.1371/journal.pone.0285395 (PMC10313032; doi:10.1371/journal.pone.0285395)
Supplement: S1 Text — (DOCX) [file pone.0285395.s001.docx]

# Interview Guide: psychosocial distress assessment

## Information

Hi, thank you for taking the time to talk to me.

- Presentation of interviewer (NJA)
- Informed consent:
  - Aim: How is mental health integrated on the hospital wards?
  - Approved by ethics committee.
  - Participation is voluntary and can be withdrawn at any time.
  - The interview is recorded and will be transcribed.
  - Data are encrypted and password-protected.
  - Only authorized persons have access to un-encrypted data and these people are bound to secrecy.
- There are no right or wrong answers, but we are interested in experiences and impressions collected during the project so far. There are also more general questions on guidelines.
- Indicate if you do not want to answer a question.
- Are there any questions?
- Sign consent
- Read the definition: “Psychosocial distress means that a patient is stressed, worried or under pressure. Often, these are difficulties related to following things: physical complaints or restrictions, emotional issues (e.g., sadness, depression, anxiety), family/children/friends, work/school, money or meaning of life/spirituality/faith.”
- Turn on audio-recorder.

## Introduction

1. Have you ever heard about the project SomPsyNet?
2. What is your role when assessing patients’ psychosocial distress, thus within the project SomPsyNet?
   1. How does the assessment of psychosocial distress look?

## Guideline Factors

1. Are the physicians and nurses familiar with the assessment of patients’ psychosocial distress?
2. Do you and your colleagues have access to information about the assessment of patients’ psychosocial distress?
3. In your opinion, are the people who developed the project SomPsyNet credible?
   1. Do you have any concerns regarding the credibility of these people? Which ones?
   2. In your opinion, do you think that the assessment of patients’ psychosocial distress is evidence-based?
4. In your opinion, is the assessment of patients’ psychosocial distress feasible?
5. In your opinion, how much effort is required to assess patients’ psychosocial distress?
   1. What makes the assessment difficult?
   2. What makes the assessment easy?
   3. Is the assessment of patients’ psychosocial distress consistent with other guidelines or recommendations you work with routinely?
   4. Can you detect any changes in patients through the assessment of psychosocial distress? In dealing with them? In patients’ well-being?
   5. In your opinion which knowledge need physicians and nurses to correctly assess patients’ psychosocial distress? Do physicians and nurses have this knowledge?
   6. What skills are needed to assess patient’s psychosocial distress? Do physicians and nurses have these skills? Are they capable to assess patients’ psychosocial distress?

## Individual health professional factors

1. How do you and your colleagues view guidelines and standardization in general?
2. Do you and your colleagues think that the assessment of patients’ psychosocial distress will lead to better outcomes? Meaning for patients, physicians/nurses, or the healthcare system?
   1. Do you and your colleagues assess patients’ psychosocial distress?
   2. What concerns about assessing patients’ psychosocial distress do you have?
   3. Do you have any concerns to assess patients’ psychosocial distress although you assess psychosocial distress? Which ones?
3. How have processes regarding patients’ psychosocial distress changed? (Who must do what different? When? Where? How? How often?)
   1. Do you have the capacity to do this?
   2. In your opinion, has the number of consultation changed? How?

## Patient factors

1. How do you perceive patients’ knowledge, needs, and expectations related to the assessment of psychosocial distress at the hospital?
   1. Do you perceive that patients have different values about the assessment of psychosocial distress than you or the recommendation? How do they differ/are similar?
   2. Have you or your colleagues any issues with assessing patients’ psychosocial distress? Why?
   3. Do you know from any issues caused by the assessment of psychosocial distress?
2. Have you ever received direct feedback from patients? What feedback?

## Professional interactions

1. Are there organizations, networks, or prevailing norms affecting the assessment of patients’ psychosocial distress?
2. Are there team or workflow issues that affect the assessment of patients’ psychosocial distress?
   1. Are changes needed in referral processes, or interactions with other systems or groups to be able to assess patients’ psychosocial distress effectively?

## Incentives and resources

1. What resources would be helpful in getting you or your colleagues to assess patients’ psychosocial distress? (financial incentives, human resources, equipment and supplies, technical capacity, patient information or others)
   1. What financial incentives do you or your colleagues, system administrators, patients, and others have to assess psychosocial distress?
   2. How does the information system facilitate or hinder the assessment of patients’ psychosocial distress?
   3. How does the quality assurance facilitate or hinder the assessment of patients’ psychosocial distress?
   4. Do you or your colleagues have the assistance you need to assess patients’ psychosocial distress? (checklists, patient information, decision aids, supervision)

## Capacity for organizational change

1. What leadership or management support do you need to assess you or your colleagues in assessing patients’ psychosocial distress?
2. In your opinion, is this support available, and do you and your colleagues know how to access it?
   1. Is the style of leadership helpful?
   2. Who supports the assessment of patients’ psychosocial distress? Who does not or supports it less?
   3. How do internal and external rules, regulations, and policies help or hinder to assess patients’ psychosocial distress?
3. How much of a priority is the assessment of psychosocial distress compared with other activities going on in your setting?
4. Is monitoring and feedback available? Would monitoring and feedback be useful?

## Social, political, and legal factors

1. What do you think why the assessment of patients’ psychosocial distress is implemented?
2. Optional: Are there any payer or funder policies that you are aware of that either help or hinder the assessment of psychosocial distress?
   1. Do influential people or groups outside the hospital help or hinder the assessment of patients’ psychosocial distress?

## Closing

1. Do you have any suggestions for improvement related to the assessment of patients’ psychosocial distress?
   1. Are there any other issues we should talk about that have not come up yet?

[turn off audio-recorder]

Again, I would like to thank you for your time. It was an interesting and informative discussion. If you have any questions or anything to add later, you can contact me. You can find my contact dates on your copy of the informed consent form.
