# Supplementary material for: Facilitators and barriers of routine psychosocial distress assessment within a stepped and collaborative care model in a Swiss hospital setting
Source: PLoS One. 2023 Jun 30;18(6):e0285395. doi: 10.1371/journal.pone.0285395 (PMC10313032; doi:10.1371/journal.pone.0285395)
Supplement: S1 Table — (DOCX) [file pone.0285395.s002.docx]

| **S1 Table** Facilitators and barriers according to the TICD domains, themes, and subthemes, illustrated by representative quotes. | | |
| --- | --- | --- |
| **Themes** | **Subthemes** | **Representative quotes** |
| **Guideline factors** | | |
| Recommendation | Quality of evidence supporting the recommendation | “Yes, I would say, the way it is tested now, no, it is gut feeling. […] So the basis why it is tested is [evidence-based], yes.” physician, age 26, female |
|  | Clarity | “I think it would be difficult, because one has to determine ‘okay, this is the definition and that has to be looked at or questioned for. This is it, I would say. If it is more gut feeling or if really determinants exist, which one would assess.” physician, age 29, female |
|  | Cultural appropriateness | I think it helps that we are a small house [hospital], because we do not have patients all the time, but maybe we have 15 [patients] a week or so. And this is far easier than 15 a day, 15 different ones.” physician, age 27, female |
|  | Accessibility of the recommendation | So far, I think, I did it [the psychosocial distress assessment] once. Just, somehow, because a nurse pointed out to me that apparently, we have to assess it [psychosocial distress] together.” physician, age 29, female |
|  | Source of the recommendation | “Yes, they [the SomPsyNet implementers] do not make an untrustworthy impression. But I feel like, one does not get into, they do not talk with us. Well, they are totally external. One thinks ‘Ah, now there is someone.’ But trustworthy, yes, definitely.” nurse, age 56, female |
|  | Consistency with other guidelines | “It is even a great addition, because we have many tools assessing the somatic aspects, but hardly any that do the mental assessment. And this makes it a great tool that complements everything, yes.” nurse, age 28, male |
| Recommended clinical intervention | Feasibility | “Really, it is simply the time. Well, it is not that it is, somehow, cognitively high demanding, but it is that I have to take the time to discuss with the patient. I have to take the time to open it up [the assessment]. I have to take the time to enter it again. Exactly, and if I have it, the time, then everybody would do it for sure.” physician, age 34, female |
|  | Accessibility of the intervention | “So what I know is that it [the assessment] appears in the curve of each patient.” physician, age 35, male |
| Recommended behavior | Compatibility | “Yes, that is actually something I pretty much always incorporate into my daily life anyway, well, if I care [for the patients], simply in the discussion.” nurse, age 38, female |
|  | Effort | “And through SomPsyNet, it is just extra work. This means I have to assess him [the patient], I have to enter it [the assessment], and this takes maybe only 2 minutes more per patient, but it is just, it sums up during everyday life. And over the days even more.” physician, age 34, female  “As said before, so it needs discussions with the patient for this assessment. We do not always have the time to do these conversations.” nurse, age 42, female |
|  | Observability | “At this time, I can say it certainly makes us somehow, one is more sensitized, but if this finally [helps] the patient – I do not know that.” nurse, age 56, female |
| **Individual health professional factors** | | |
| Knowledge and skills | Domain knowledge | “Well, it is simple, because we simply notice that patients – well some patients – they come to the hospital over and over again. Simply because there are many, many factors that they [the patients] can no longer manage at home. But these are not necessarily reasons for hospitalization.” nurse, age 42, female |
|  | Awareness and familiarity with the recommendation | “And organizational I would say it is very difficult for physicians, because the SomPsyNet project was presented once or twice or three times on our ward. And the nurses have actually a very constant team. This means that we actually all know what theoretically SomPsyNet is. But physicians, they change about all four weeks. This means that the generation of physicians we have here does actually not even know that SomPsyNet exists.” nurse, age 28, male |
|  | Knowledge about own practice | “I think, probably sometimes one thinks that this [the assessment] maybe has no consequence. So, I think if a patient is well, then one thinks ‘yes, he/she [the patient] is probably not or only distressed a bit and it would probably not help much if this is further assessed’.” physician, age 31, male |
|  | Skills needed to adhere | “You cannot generalize. This is not as simple. But I think we are very empathetic and therefore, we are popular. Because we can simply work on a more personal level and you can see that in the way we deal with each other.” physician, age 27, female |
| Cognitions | Agreement with the recommendation | “Hmm, as said before, for me it is not yet tangible. It is…yes, I have to be convinced of the tool. Also that I complete it with full consciousness, and that I know what I do. And now, this is just my insecure clicking around based on gut feeling. Well, just casually said.” nurse, age 29, male |
|  | Attitudes towards guidelines in general | “Well, in principle I like any regulations. But I think I was and I still am not a fan of over-regulations, because afterwards there is no scope and there are always problems in medicine that are not white or black – it is always grey.” physician, age 26, male |
|  | Expected outcome | “Well, I do think that as soon as the study phase is over, one can take action and one can identify reasons, and one can prevent rehospitalizations. Yes, then, also patients’ quality of life increases if one can find suitable actions or projects at home.” nurse, age 46, female |
|  | Intention and motivtation | “Well, I do it, because it has to be done. But I do not further think about how important it [the assessment] is. I just do it, because it has to be done. But I think, if I know more about it or if I have more information, I would take it more serious.” nurse, age 35, female |
|  | Self-efficacy | “I am just wondering if I am correct in my assessment? So did I assess correctly? Or am I wrong? Well, this is the only question I have. I do not want to give a very bad or very positive assessment although it is not the case. I do not want to miss a problem.” nurse, age 42, female |
|  | Learning style | “I think there was someone here where you could go [to the training] and unfortunately, I missed it. I would have preferred that, but yes, it is my own fault.” nurse, age 38, female  Well, one has to demonstrate it to me – that is the best way I learn. If there are clips that explain this well, yes.” nurse, age 35, female |
|  | Emotions | “Yes, the more I am distressed, the worse I make the assessment.” physician, age 34, female |
| Professional behaviour | Nature of the behaviour | “[…] and that we can then once make a general assessment of the psychosocial distress of the patient during hospitalization. And this I do with a click on a scale, which, I think, is from zero to ten or green to red. And the assessment is relatively intuitive, is he [the patient] distressed or not and I just make it, one click, yes.” physician, age 35, male |
| **Patient factors** | | |
| Patient needs |  | “For many it is important. For some it is important that one addresses it [psychosocial distress]. For others, no, they want to be left alone. Many. They say that nobody can do anything about it.” nurse, age 35, female |
| Patient beliefs and knowledge |  | “Well, I do not know if they [patients] even are aware that this [psychosocial distress assessment] is going on. We just do it independently. We do not bring up that this exists. We just tick it.” physician, age 26, female |
| Patient motivation |  | “It is important to them to hand over the survey or what they have to complete. This is very important to them that someone is coming. We notice that.” nurse, age 46, female |
| Patient behaviour |  | “Well, I think some patients can express relatively well what they need and whether they think that they can profit. But for many patients, one does not know. One has to offer that a bit empirically. And sometimes it works and sometimes not at all.” physician, age 31, male  “And I think it is only easy with extrovert personalities, if they just tell you that there is something else and that is why they [the patients] are nervous. There are people who very quickly confide very personal things, but mostly that is not the case.” nurse, age 49, female |
| **Professional interactions** | | |
| Communication and influence |  | “And maybe, if the physician decided in advance, I think one adapts automatically a bit. This means physicians to nurses and the other way around. I think one orients oneself a bit if one does not know exactly [how to assess the patient]. Well, before one omits it, one orients oneself on the person who assessed in advance.” nurse, age 29, male |
| Team processes |  | “At the hospital, we have many interprofessional discussions and there, many things are done and one discusses about it [the distress of patients]. Each patient has, I do not know, five or ten minutes a week. And I believe, it is important to have a common goal for the patient.” nurse, age 23, female |
| Referral processes |  | “Well, we all know the system and we know how to trigger consultations – this is actually clear. And I think we do not have to write a lot for consultations.” physician, age 29, female |
| **Incentives and resources** | | |
| Availability of necessary resources |  | And that is why I think that the resources like the holistic care, having time for the entire human, are no longer a goal. And from that, it is not simply a resource, a technical mean or as said before, what would help me.” nurse, age 49, female |
| Financial incentives and disincentives |  | “I only can imagine if there is actually a psychosocial problem leading to a hospitalization and if one can somehow solve it sustainably that costs could be reduced.” physician, age 26, female |
| Nonfinancial incentives and disincentives |  | “Yes, it is more that we did not really received a training. It is more ‘do it now’, without any background information except the sentence that comes with the assessment explaining what it is about. And this makes it a bit difficult for me. Yes, probably, it would have been awesome to have a short – a quarter – information what it is about. So that one knows a bit what to look out for. Just a bit what the background thoughts are or what is planned with it [the assessment]. We did not learn about all of this.” physician, age 26, female  “[…] a year ago, we heard about it. And then, we had a second meeting where it was explained how we should do that, why it [the assessment] should be done, what the goal is and why it is done.” nurse, age 42, female |
| Information system |  | “[…] I would like to see the score or if it is done at all. Because in our case, it displays only SomPsyNet and that is it. And then, one does not know is it done, is it not done, is it a good score, is it a bad score.” nurse, age 27, female |
| Quality assurance and patient safety system |  | “Well, neither nor. I did not associate it [the assessment] with quality assurance.” nurse, age 56, female |
| Continuing education system |  | “Yes, maybe it would be great to again have an input of the study team who comes again and maybe tell again what it actually is about and how important it is. And just revive.” nurse, age 38, female |
| Assistance for clinicians |  | “I feel like this assessment with a scale from zero to ten is a bit difficult, because what means ten and what means zero? And it is very personal and I do not know if maybe two/three questions would be better, because then, it would not be so – it is very personal, as I said before.” nurse, age 23, female |
| **Capacity for organizational change** | | |
| Capable leadership |  | “Well, sure our management who for sure affects it [SomPsyNet] positively, because she fully support it and if she has information, it is always in our weekly information. And she said now and then ‘remember the SomPsyNet project! If you have any questions, pass by at any time.’ Or if something happened, she always took care of it.” nurse, age 28, male |
| Relative strength of supporters and opponents |  | “Also, it [SomPsyNet] is promoted and desired by the house [hospital]. This makes one feel to do something important.” nurse, age 46, female |
| Priority of necessary change |  | Honestly, a very low one [priority]. Because, in fact, it is all according to the urgency. Well, if somehow the medication is not administered, then I have the consequences, so I will administer it [the medication] 100%. If I do not document it, then everybody sees it and the nursing process cannot be guaranteed, so I will do it for sure. If I do not document SomPsyNet, nothing happens at all and that is why, so to say, if one looks at the ranking, it is pretty much the lowest one on the hierarchy that has to be completed. Yes, because it just does not have any consequences, direct ones, on the daily routine.” nurse, age 28, male |
| Monitoring and feedback |  | “Yes, she always say that it is good. But generally, for everybody. She thinks it is great that everybody completes it always. And yes, if it is not completed, she calls to ask that one does it.” nurse, age 35, female |
| **Social, political and legal factors** | | |
| Influential people |  | Well, I do not know, but I think, in politics, of course, this is something exciting, because they are maybe interested in reducing hospitals and costs. Without really having the wish in terms of content, one thinks that such a project is still promising.” nurse, age 49, female |
